# Performance of the HIV Blot 2.2, INNO-LIA HIV I/II Score, and Geenius HIV 1/2 Confirmatory Assay for use in HIV confirmation

**DOI:** 10.1371/journal.pone.0199502

**Published:** 2018-06-21

**Authors:** Chui Ching Wong, Siew Hoon Lim, Chai Teng Tan, Sook Yin Lui, Yee Leng Lee, Kwai Peng Chan

**Affiliations:** 1 HIV Reference Laboratory, Virology Laboratory, Department of Microbiology, Division of Pathology, Singapore General Hospital, Singapore, Singapore; 2 Duke-NUS Medical School, Singapore, Singapore; The University of Hong Kong, HONG KONG

## Abstract

In view of recent revised recommendations for human immunodeficiency virus (HIV) confirmatory testing, the performance of 3 HIV confirmatory assays was compared. Using the HIV Blot 2.2 (MP-WB), the INNO-LIA HIV I/II Score (INNO), and the Geenius HIV 1/2 Confirmatory Assay (Geenius), we tested 199 HIV-1 positive, 161 HIV negative, 65 HIV western blot indeterminate, 26 HIV seroconversion, 34 early HIV infection and 4 HIV-2 positive archived specimens. We show that all 3 assays had comparable test sensitivity in the detection of HIV-1 positive cases. However, less non-specific reactivity was observed with the INNO and Geenius assays, where both of them were able to resolve MP-WB indeterminate cases. When early HIV cases were considered, INNO and Geenius were more likely to confirm an early-stage infection as positive. Nevertheless, overall poor sensitivity (25.5% - 44.7%) of these assays for the detection of early cases was observed, likely because these cases had very low or non-detectable levels of HIV antibodies. Hence, further testing by a nucleic acid test or a p24 antigen test of specimens reactive on screening with a fourth generation Ag/Ab assay that are negative on confirmatory testing for HIV-specific antibody, may be useful. In conclusion, INNO and Geenius had comparable test performance, although the ease of use and shorter assay time for Geenius may make it the preferred choice for laboratories. In that regard, of note is our observation of non-specific reactivity of lipaemic specimens to the HIV-2 gp140 band in the Geenius assay, which should prompt caution when interpreting results of such specimens.

## Introduction

In recent years, revised algorithms for Human Immunodeficiency Virus (HIV) confirmatory testing have been recommended by various institutions worldwide. The Centers for Disease Control and Prevention (CDC) in the United States (US) updated its recommendations for HIV confirmation in 2014, such that after an initial fourth-generation HIV Ag/Ab screening test, the use of HIV western blot was replaced by a HIV-1/HIV-2 antibody differentiation assay as the supplemental test for HIV confirmation [1]. In 2015, the World Health Organization (WHO) reiterated its earlier recommendation that in settings where the HIV prevalence in the population tested is greater than 5%, a diagnosis of HIV-positive should be issued to individuals with 2 sequential reactive tests. However, if the HIV prevalence in the population tested is less than 5%, a diagnosis of HIV-positive should be issued to individuals with 3 sequential reactive tests to enhance the positive predictive value [2–3]. On a similar note, HIV screening and confirmation guidelines in the United Kingdom (UK) published by Public Health England (PHE) also recommended 2 separate reactive fourth-generation HIV Ag/Ab screening tests for HIV screening, followed by a HIV-1/HIV-2 antibody differentiation immunoassay or a further immunoassay and then a HIV-1/HIV-2 typing assay for HIV confirmation [4]. As regards Australia, the Public Health Laboratory Network recommends that repeatedly reactive HIV antibody or antigen/antibody cases should be confirmed using a supplemental assay, inherently a western blot or a line probe immunoassay, with use of a nucleic acid or antigen test as necessary [5].

In the revised recommendations from the US and UK, a HIV-1/HIV-2 differentiation assay has been included to allow diagnosis of HIV-2 infection as the virus has an intrinsic resistance to non-nucleoside reverse transcriptase inhibitors as well as to some protease inhibitors [6]; to use tests that detect only HIV-1 may cause HIV-2 cases to be missed [7–8]. The revised algorithms will allow appropriate treatment according to the HIV type causing the infection.

Singapore has a low prevalence of HIV, with an exceedingly low prevalence of HIV-2. The latest national statistic shows that in 2016, 103.7 per million resident population were diagnosed with HIV infection [9]. Our laboratory confirms the status of all samples screened reactive for HIV in the country. We use the triple test algorithm, as recommended by WHO [3] and similar to Australia, which also has a low HIV-2 prevalence [10], the western blot is employed as one of the confirmatory tests. Specifically, we use the HIV Blot 2.2 Western Blot assay (MP Biomedicals Asia Pacific, Singapore) which includes a HIV-2 specific peptide, reactivity to which will prompt follow-up testing using a HIV-2 confirmatory assay. Results are interpreted according to the manufacturer’s instructions and HIV-2 cases will be reported accordingly. In the past decade, only 3 HIV-2 positive cases have been reported by our laboratory, but none of them was a Singapore resident.

In consideration of the recent recommendations for HIV-1/HIV-2 differentiation, a study was carried out to compare the performance of the HIV Blot 2.2 Western Blot assay (MP-WB) to the INNO-LIA HIV I/II Score (INNO, Fujirebio, Ghent, Belgium) and the Bio-Rad Geenius HIV 1/2 Confirmatory Assay (Geenius, Bio-Rad, CA, US), for use in HIV confirmation to detect antibodies to HIV-1 and HIV-2. Briefly, the MP-WB assay is an enzyme immunoassay where separated HIV-1 viral lysate antigens and a specific HIV-2 synthetic peptide are incorporated along a nitrocellulose strip. With the INNO assay, which is another enzyme immunoassay, recombinant proteins and synthetic peptides from HIV-1 and HIV-2 are coated on a nylon strip. In both MP-WB and INNO assays, specific antibodies to HIV-1 and HIV-2, if present in a sample, will bind to the HIV-1 and HIV-2 antigens on the strip. Following a wash step to remove unbound materials, antibodies that bind specifically to HIV proteins can be visualized after further reactions with anti-human IgG conjugated with alkaline phosphatase and an enzyme substrate. The Geenius assay is a lateral flow immunochromatographic assay where HIV-1 and HIV-2 recombinant antigens are bound to the solid phase membrane in a test cassette. In sequential steps, the sample migrates to the test strip where specific anti-HIV antibodies that are present in the sample are captured by the immobilized antigens in the test area. The addition of buffer facilitates the release of conjugated colloidal gold protein A which binds to the captured antibodies enabling the antibody bands to be visualized.

## Materials and methods

Specimens except for the HIV-2 positive ones were anonymized clinical specimens archived after HIV confirmation testing using a fourth generation HIV Ag/Ab screening assay (ARCHITECT HIV Ag/Ab Combo assay, Abbott Laboratories, IL, US) [11–12], and 2 other HIV antibody assays, the SERODIA-HIV1/2 particle agglutination assay (PA, Fujirebio, Ghent, Belgium) and the MP-WB assay. The HIV-2 positive specimens were external proficiency test samples without patient identifiers. As defined below, the test panel consisted of 489 specimens across 6 categories, including 199 HIV-1 positive, 161 HIV negative, 65 HIV western blot indeterminate, 26 HIV seroconversion (13 pairs), 34 HIV antigen positive (p24 antigen positive) and 4 HIV-2 positive specimens (Table 1). Ethics review waiver (reason for waiver: research involves analysis of samples without identifiers) was given by the SingHealth Centralised Institutional Review Board (reference number: 2017/2534).

**Table 1:** List of specimens used.

Categories	No. of specimens	Definitions^1^
HIV-1 Positive	199	Tested reactive to HIV Screen, and positive forHIV-1 using PA and MP-WB.
HIV Negative	161	Tested non-reactive to HIV Screen, and negative for HIV using PA and MP-WB.
HIV Indeterminate	65	Tested indeterminate to HIV, showing reactivity to HIV antigen bands^2^ by MP-WB with no seroconversion.
HIV Seroconversion^3^	26 (13 pairs)	Paired serum specimens showing seroconversion. 1^st^ specimen indeterminate to HIV by MP-WB, 2^nd^ specimen positive to HIV by MP-WB.
HIV Antigen Positive^3^	34^4^	Tested reactive to HIV screen and positive for HIV p24 antigen.
HIV-2 Positive	4	External proficiency test specimens verified to be positive to HIV-2.

^1^HIV Screen, Abbott ARCHITECT HIV Ag/Ab Combo (Abbott Laboratories, IL, US); PA, Serodia HIV I/II particle agglutination (Fujirebio, Ghent, Belgium); MP-WB (MP Biomedicals HIV Blot 2.2 Western Blot assay).

^2^Non-specific bands to p24 alone (n = 57), p31 alone (n = 1), gp160 alone (n = 2), p17 and p24 (n = 2), p24 and p66 (n = 1), and, p24 and gp160 (n = 2), were observed.

^3^The HIV seroconversion and HIV antigen positive categories were mutually exclusive. The first samples of seroconversion pairs were HIV IND by MP-WB but were negative for HIV antigen. HIV antigen-positive samples were not included in the seroconversion group.

^4^Samples were negative or weakly reactive (± or 1+) by PA, not positive by MP-WB, and positive for HIV antigen using the HIV VIDAS DUO ULTRA (bioMérieux, Marcy-l'Étoile, France) indicating early HIV infection. Specimens were either confirmed positive for HIV p24 using a HIV p24 neutralization assay (VIDAS HIV P24 II Confirmation, bioMérieux, Marcy-l'Étoile, France), or had follow-up specimens that were positive for HIV RNA using the COBAS® TaqMan® HIV-1 Test version 2.0 (Roche Diagnostics GmbH, Mannheim, Germany) or for HIV-1 antibody by MP-WB.

HIV-1 positive specimens were screened reactive to HIV and positive for HIV-1 antibodies using PA and MP-WB. Negative specimens on the other hand, were screened non-reactive to HIV and negative for HIV antibodies using PA and MP-WB. Specimens in the western blot indeterminate category were screened reactive to HIV, tested negative to HIV using PA, and showed reactivity to HIV antigen bands (in particular, p24) by MP-WB that was insufficient for a positive profile according to the manufacturer. For these cases, no seroconversion ensued on subsequent specimens collected at least 3 months after. Conversely, seroconversion pairs were paired specimens where the first sample was screened reactive, was either negative, weakly reactive, or positive by PA, and tested indeterminate to MP-WB, while the second sample was confirmed to be positive for HIV-1 as determined above. Early HIV infection specimens were those screened reactive for HIV, either negative or weakly reactive by PA, not positive by MP-WB, but positive for HIV p24 antigen using the HIV VIDAS DUO ULTRA (bioMérieux, Marcy-l'Étoile, France). These specimens were either confirmed positive for HIV p24 using a HIV p24 neutralisation assay, the VIDAS HIV P24 II Confirmation (bioMérieux, Marcy-l'Étoile, France), or had follow-up specimens that were positive for HIV RNA using the COBAS® TaqMan® HIV-1 Test version 2.0 (Roche Diagnostics GmbH, Mannheim, Germany) or for HIV-1 antibody by MP-WB. The HIV seroconversion and HIV antigen positive categories were mutually exclusive. HIV-2 specimens were external proficiency test specimens verified to be positive for HIV-2.

The manufacturer’s instructions on test procedures and interpretations were followed for all 3 assays studied. The MP-WB assay was performed on the automated system Autoblot 20 and blots were read and graded manually by three users. The INNO assay was performed on the automated system Auto-LIA 48 (Fujirebio, Ghent, Belgium) and the assay strips were read and graded using the LiRAS software (Fujirebio, Ghent, Belgium). The Geenius assay was performed manually and then read and graded using the Geenius Reader and Software (Bio-Rad, CA, US).

Statistical analyses and calculation of the Cohen’s kappa coefficient were performed using the MedCalc Statistical Software (version 18.2.1, MedCalc Software, Ostend, Belgium).

## Results

Results for 489 specimens tested by MP-WB, INNO and Geenius were compared (Table 2). All 199 HIV-1 positive cases, previously tested positive for HIV-1 by MP-WB, were also positive for HIV-1 using INNO and Geenius. Of the 161 HIV negative cases, 157 cases were tested negative by all 3 assays. The remaining 4 samples were tested negative using MP-WB, and 2 each were tested indeterminate and HIV-2 indeterminate by INNO and Geenius, respectively (Table 2). As these cases were also non-reactive for HIV using the screening and particle agglutination assays, the indeterminate results were likely due to non-specific reactivity. These findings indicate that INNO and Geenius were as sensitive as MP-WB in detecting HIV-1 positive cases (test sensitivity: 100%, Table 3), and test specificity was also comparable (98.8%, Table 3). The positive and negative predictive values (99.0% and 100%, respectively, Table 3) were identical for both INNO and Geenius.

**Table 2:** Test results using the MP Biomedicals HIV Blot 2.2 assay, Fujirebio INNO-LIA HIV I/II Score, and Bio-Rad Geenius HIV 1/2 Confirmatory Assay^1^.

Test Category	MP Biomedicals HIV Blot 2.2^2^					Fujirebio INNO-LIA HIV I/II Score^2^				Bio-Rad Geenius HIV 1/2 Confirmatory Assay^2^					
	POS	POS (HIV-2)	NEG	IND	IND (HIV-2)	HIV-1 POS	HIV-2 POS	NEG	IND	HIV-1 POS	HIV-2 POS	NEG	HIV-1 IND	HIV-2 IND	IND
HIV-1 Positive	199	-	-	-	-	199	-	-	-	199	-	-	-	-	-
HIV Negative	-	-	161	-	-	-	-	159	2^3^	-	-	159	-	2^3^	-
HIV Indeterminate	-	-	-	65	-	-	-	60	5	-	-	64	1^4^	-	-
HIV Seroconversion—First Sample	-	-	-	13	-	9	-	1	3	6^5^	-	4	3	-	-
HIV Seroconversion—Second Sample	13	-	-	-	-	13	-	-	-	13	-	-	-	-	-
HIV Antigen Positive	-	-	26	8	-	-	-	29	5	1	-	31	2	-	-
HIV-2 Positive	-	1	-	-	3^6^	-	4	-	-	-	4	-	-	-	-

^1^ Fleiss’ multiple raters generalized kappa coefficient for all 3 assays, k [95% CI] = 0.781 [0.734–0.827]. Cohen’s kappa coefficient for MP-WB and INNO k [95% CI] = 0.722 [0.674–0.771], MP-WB and Geenius k [95% CI] = 0.711 [0.662–0.759], and INNO and Geenius k [95% CI] = 0.933 [0.903–0.964]. The coefficients were calculated using HIV Positive, HIV Negative and HIV Indeterminate as 3 result groups. Geenius HIV-1 indeterminate and HIV-2 indeterminate samples were considered as HIV Indeterminate.

^2^ POS—Positive; POS (HIV-2)—Positive with HIV-2 indicated; NEG—Negative; IND—Indeterminate; IND (HIV-2)–Indeterminate with HIV-2 indicated; HIV-1 POS–HIV-1 Positive; HIV-2 POS–HIV-2 Positive, HIV-1 IND–HIV-1 Indeterminate; HIV-2 IND–HIV-2 Indeterminate.

^3^The 2 INNO indeterminate and 2 Geenius HIV-2 indeterminate cases were 4 separate cases

^4^The Geenius HIV-1 indeterminate case was also INNO indeterminate

^5^All 6 Geenius HIV-1 positive cases were also HIV-1 positive using INNO

^6^Cases were confirmed as HIV-2 positive upon further testing according to the manufacturer’s guidelines.

**Table 3:** Performance of the Fujirebio INNO-LIA HIV I/II Score and Bio-Rad Geenius HIV 1/2 Confirmatory Assay.

Test Performance^1^	Fujirebio INNO-LIA HIV I/II Score	Bio-Rad Geenius HIV 1/2 Confirmatory Assay
Test Sensitivity^2^ [95% CI]	100% [98.2–100.0] (203/203)	100% [98.2–100.0] (203/203)
Test Specificity [95% CI]	98.8% [95.6–99.9] (159/161)	98.8% [95.6–99.9] (159/161)
Positive predictive value^3^	99.0% [96.2–99.8]	99.0% [96.2–99.8]
Negative predictive value^3^	100%	100%

^1^Test performance for INNO and Geenius were compared using results from MP-WB as a reference.

^2^HIV positive cases include HIV-1 positive and HIV-2 positive (total: 203 cases)

^3^Negative MP-WB samples that were tested indeterminate by INNO and Geenius were considered as HIV “false positive” by INNO and Geenius in the predictive value calculations.

Regarding HIV indeterminate cases where there was no seroconversion for at least 3 months, 60 of 65 cases were tested negative by both INNO and Geenius. As for the remaining 5 cases, 1 was INNO indeterminate and Geenius HIV-1 indeterminate, while 4 were INNO indeterminate and Geenius negative (Table 2). Although the results of HIV negative cases seem to indicate that INNO and Geenius had comparable test specificity with MP-WB, the findings of HIV indeterminate cases suggest that in contrast to MP-WB, non-specific binding of serum samples to the HIV antigen bands in the INNO and Geenius assays occurred less often, resulting in the assays being more specific than MP-WB.

A total of 13 HIV seroconversion pairs were included in the evaluation. These specimens were selected based on results from MP-WB where the patient had an indeterminate result on the first sample and a positive HIV result on the second. Among the first specimens of the seroconversion pairs, 69.2% (9/13) and 46.2% (6/13) were tested HIV-1 positive using INNO and Geenius, respectively (Table 2). Of note, 6 of these specimens were positive using both INNO and Geenius (Table 4). Table 4 lists the breakdown of results for all early cases. The remaining specimens were either negative (INNO, n = 1; Geenius, n = 4), indeterminate (INNO, n = 3) or HIV-1 indeterminate (Geenius, n = 3, Tables 2 and 4). The second samples of all 13 seroconversion samples were tested positive to HIV by INNO and Geenius. HIV antigen positive specimens (n = 34) were tested negative (n = 26) or indeterminate (n = 8) using MP-WB, while INNO tested 29 negative and 5 indeterminate cases (Table 2). There were 1 HIV-1 positive, 31 HIV negative and 2 HIV-1 indeterminate cases when Geenius was used (Tables 2 and 4). Notably, 23 antigen-positive specimens were tested negative by all 3 assays (Table 4).

**Table 4:** Breakdown of results for early HIV cases.

Test Category	No. of Samples	MP Biomedicals HIV Blot 2.2^1^^,^^2^		Fujirebio INNO-LIA HIV I/II Score^1^^,^^2^			Bio-Rad Geenius HIV 1/2 Confirmatory Assay^1^^,^^2^		
		NEG	IND	HIV-1 POS	NEG	IND	HIV-1 POS	NEG	HIV-1 IND
HIV Seroconversion—First Sample	6		x	x			x		
	3		x	x				x	
	2		x			x			x
	1		x			x		x	
	1		x		x				x
HIV Antigen Positive	1		x		x		x		
	1		x			x			x
	1		x			x		x	
	5		x		x			x	
	1	x				x			x
	2	x				x		x	
	23	x			x			x	

^1^The symbol “x” indicates respective result for MP Biomedicals HIV Blot 2.2 assay, Fujirebio INNO-LIA HIV I/II Score, and Bio-Rad Geenius HIV 1/2 Confirmatory Assay. Result fields are omitted if there are no samples in that result group.

^*2*^ NEG–Negative; IND–Indeterminate; HIV-1 POS–HIV-1 Positive; HIV-1 IND–HIV-1 Indeterminate.

Taking the first specimen of the seroconversion pairs and the antigen positive specimens into consideration, INNO (19.1%, 9/47) and Geenius (14.9%, 7/47) were more likely to confirm an early-stage infection as positive when compared to MP-WB (0%, 0/47; INNO vs MP-WB, p<0.05; Geenius vs MP-WB, p<0.05). On the other hand, it was observed that MP-WB was able to detect more cases of early-stage infection as indeterminate or positive (44.7%, 21/47) as compared to Geenius (25.5%, 12/47, p = 0.05) and INNO (36.2%, 17/47, p = 0.40), although results were not statistically significant. When the early cases were considered with the HIV-positive specimens, 81.2% (95% CI: 75.8–85.9, 203/250), 84.8% (95% CI: 79.7–89.0, 212/250) and 84.0% (95% CI: 78.9–88.3, 210/250) of the cases were tested positive using MP-WB, INNO and Geenius, respectively.

With regard to the 4 HIV-2 positive specimens, using MP-WB, 1 specimen was HIV-1 positive with HIV-2 indicated while the remaining 3 specimens were HIV indeterminate with HIV-2 indicated. In accordance with the manufacturer’s recommendation, further testing for HIV-2 was performed for the specimens and all 4 specimens were confirmed to be HIV-2 positive. Using either INNO or Geenius, all 4 specimens were HIV-2 positive.

There was substantial test agreement among the 3 assays across all specimens tested (Fleiss’ kappa, *k* = 0.781, Table 3) [13–14], and the inter-rater agreement between INNO and Geenius was almost perfect (Cohen’s kappa, *k* = 0.933, Table 3) [14–15]. Notably, although there was substantial inter-rater agreement between MP-WB and INNO (*k* = 0.722), and MP-WB and Geenius (*k* = 0.711), the co-efficients were lower than that of INNO and Geenius, mainly due to the MP-WB indeterminate specimens that were resolved as negative using INNO and Geenius.

In the course of the evaluation, it was noted that 5 negative samples had detectable p31 (n = 1) or gp140 (n = 4) bands when tested using Geenius, but were tested negative using MP-WB or INNO. Interestingly, these samples were lipaemic and upon re-testing with Geenius after a centrifugation step at 11,000 x *g* for 10 mins, 3 of the results became negative. However, 2 cases still had detectable gp140 bands and were considered ‘HIV-2 indeterminate’.

## Discussion

Previous studies compared the performance of Geenius to MP-WB [16–17] and the performance of Geenius to INNO separately [18–20]; only one study compared the performance of all 3 assays on 16 samples to stage HIV recent infection [21]. This is the first expanded study on 489 specimens to compare them together for use in HIV confirmation, where we show that INNO and Geenius assays have comparable performance to MP-WB for confirming HIV-positive and HIV-negative cases. Additionally, INNO and Geenius were able to resolve most (92.3%, 60/65 and 98.5%, 64/65, respectively) of the MP-WB HIV indeterminate cases that did not seroprogress as being negative to HIV. While this suggests higher specificity of INNO and Geenius assays, one limitation of this study is that specimens were selected based on results from MP-WB and this may bias against the latter assay.

On the presumption that low levels of antibodies to HIV may be present and may be detectable, early HIV cases (the first specimen of seroconversion pairs and antigen positive specimens), were also tested. Results showed that INNO and Geenius (19.1% and 14.9%, respectively) were more likely to confirm an early-stage infection than MP-WB (0%), while the latter was able to detect more cases as indeterminate in the early-stage. This suggests that the MP-WB indeterminate results can provide a good initial indication of whether a patient is infected and highlight cases for nucleic acid or p24 antigen detection, or to be followed-up to be subsequently confirmed as positive. On the other hand, if the first samples were tested negative as happened in some INNO and Geenius cases, it is possible that they may not be investigated further depending on the algorithm used, thereby losing the opportunity for diagnosis of HIV infection. Whichever of these 3 confirmatory assays is used, the proportion of early cases identified remains low (25.5% - 44.6%). Further testing by a nucleic acid test or a p24 antigen test of specimens reactive on screening with a fourth generation Ag/Ab assay that are negative on confirmatory testing may be useful. The inclusion of a nucleic acid test on such samples is in the revised recommendations by both CDC [1] and PHE [4]. In low HIV prevalence regions that adopt the triple test algorithm recommended by WHO [3], the third assay can be one that tests for nucleic acid or antigen, resources permitting, to aid in the diagnosis of early cases.

When comparing INNO and Geenius, our findings showed very good test agreement (Cohen’s kappa = 0.933) and test specificity (98.8%). Although this corroborated with 2 separate studies that reported comparable test specificity for both INNO and Geenius [18–19], specimens in those studies were selected based on INNO results, which could have introduced a bias against the INNO assay. In this study, specimens were selected based on MP-WB results, allowing a fair comparison between INNO and Geenius to be made. Notwithstanding the similar test specificity for INNO and Geenius, as a rapid test, the Geenius assay offers a simpler test methodology and shortened hands-on time which may be more advantageous for laboratories since it reduces both the time to training the operator and to reporting results [21].

With the recent introduction of the Geenius assay, studies have shown that incorrectly reported “indeterminate” but true negative cases using either MP-WB [16] or INNO [18] could be resolved by Geenius, suggesting increased test specificity for the Geenius assay. However, the question then arises whether Geenius “indeterminate”, true negative cases can also be resolved by the other assays. Our study revealed some non-specific reactivity in the Geenius assay, especially to HIV-2 and particularly for lipaemic specimens, that could be resolved as negative by both INNO and MP-WB. Notably, in CDC’s test guidance for Geenius users, repeat testing of HIV-2 indeterminate and HIV indeterminate specimens was also recommended [22]. As the Geenius assay is designed to discriminate between HIV-1 and HIV-2, these observations suggest that the kit could possibly give rise to spurious HIV-1/HIV-2 differentiation results. As more laboratories use Geenius for HIV confirmation, future evaluations can be performed to ascertain its performance.

The ability to differentiate HIV-1 and HIV-2 infections was one of the key purposes for the revised algorithms from CDC and PHE [1, 4]. In this study, we show that when HIV-1 positive western blot specimens were tested using a HIV 1/2 differentiating test like INNO or Geenius, none of the specimens was HIV-2 positive. Similar findings were also observed in a separate study where 36 samples were tested in parallel by MP-WB and INNO (Lim SH and Chan KP, unpublished). Contrary to reports that HIV western blot failed to detect HIV-2 [7–8], our experience with the MP-WB has been the successful identification of all HIV-2 positive cases, admittedly a small number, for further HIV-2 testing. The difference could lie with the inclusion of a HIV-2 specific synthetic peptide band in the MP-WB test, which is absent from the western blot kits used in other studies [7–8]. In the case of the latter where the HIV-1 western blot assays are not able to detect HIV-2 infections [23], a HIV 1/2 differentiation test would be indicated in the testing algorithm, if the burden of HIV-2 infection in the population is of concern. On the contrary, in countries where the prevalence of HIV-2 is very low, and where INNO or Geenius costs considerably more than non-differentiating assays, the inclusion of a HIV 1/2 differentiating assay in the HIV testing algorithm may not confer significant benefit especially if the western blot, long held to be the standard for confirming HIV results, is able to flag HIV-2 cases for confirmation. As such, we see continued utility of the MP-WB assay in our Singapore population of low HIV burden and negligible HIV-2 prevalence. The HIV 1/2 differentiation test can be used judiciously to supplement the MP-WB assay, in the scenarios where the results suggest that HIV-2 is indicated, or where indeterminate result profiles suggest they could be resolved with the use of a differentiation assay. In this instance, the Geenius assay would likely be more useful because of its ease of use and lower cost compared to the INNO assay in our local context.

## Conclusion

In conclusion, our study showed comparable test sensitivity and test specificity for HIV-1 for all 3 assays. The 2 HIV 1/2 differentiating assays, INNO and Geenius, were able to resolve MP-WB HIV indeterminate cases without seroprogression as being negative to HIV, and were also able to confirm more early-stage cases as positive. MP-WB, however, was able to detect more early cases as indeterminate. Despite the comparable test performance of INNO and Geenius, Geenius may have an advantage over INNO because of its simpler and shorter test procedure. Test results, however, should be interpreted with caution for lipaemic samples tested with Geenius, particularly when there is reactivity to HIV-2 bands. The overall test sensitivity for early-stage infections was low for all 3 antibody-based assays and nucleic acid or p24 antigen testing would probably be useful for diagnosis of such cases. In our opinion, MP-WB still has a place in the laboratory diagnosis of HIV if the HIV-2 prevalence is very low. Its use can be supplemented with that of a differentiating assay when indicated. Additionally, parallel testing can be performed in future for equitable assessment of MP-WB with the other 2 assays as samples in this study were categorized on the basis of results with MP-WB.
